# Laparoscopic Roux-en-Y gastric bypass in a patient with situs inversus totalis: case report

**DOI:** 10.1093/jscr/rjac336

**Published:** 2022-07-22

**Authors:** Pablo Gerardo Zorrilla-Blanco, Gerardo Muñoz-Maldonado, Luis Fernando Zorrilla-Nuñez, Noé Núñez-Jasso, Fernando González-Zorrilla, Lilia Andrea Mata-De Anda, Manuel de la O-Escamilla

**Affiliations:** Department of General Surgery, Hospital Universitario “Dr. José Eleuterio González”, Universidad Autónoma de Nuevo León, Monterrey, Nuevo León, México; Department of General Surgery, Hospital Universitario “Dr. José Eleuterio González”, Universidad Autónoma de Nuevo León, Monterrey, Nuevo León, México; Department of General Surgery, Hospital Universitario “Dr. José Eleuterio González”, Universidad Autónoma de Nuevo León, Monterrey, Nuevo León, México; Department of General Surgery, Hospital Universitario “Dr. José Eleuterio González”, Universidad Autónoma de Nuevo León, Monterrey, Nuevo León, México; Department of General Surgery, Hospital Universitario “Dr. José Eleuterio González”, Universidad Autónoma de Nuevo León, Monterrey, Nuevo León, México; Department of General Surgery, Hospital Universitario “Dr. José Eleuterio González”, Universidad Autónoma de Nuevo León, Monterrey, Nuevo León, México; Department of General Surgery, Hospital Universitario “Dr. José Eleuterio González”, Universidad Autónoma de Nuevo León, Monterrey, Nuevo León, México

**Keywords:** situs inversus, bariatric surgery, gastric bypass, laparoscopic

## Abstract

The purpose of this report is to present the case of a patient with situs inversus totalis (SIT) who underwent laparoscopic gastric bypass and review of the literature. Situs inversus is a rare congenital abnormality that occurs in ~0.01% of the population and is characterized by transposition of the abdominal viscera1. When associated with dextrocardia, it is known as SIT. Electrocardiography reveals a reversal of the electrical waves of the heart and is the diagnostic measure of choice. Roux-en-Y laparoscopic gastric bypass for morbid obesity is one of the most effective bariatric procedures currently considered as the gold standard in bariatric surgery. We present the case of a 19-year-old male with morbid obesity and diagnosis of SIT, who underwent a laparoscopic gastric bypass adjusting the technique to the anatomical changes typical of this variant. It can be concluded, based on the previously compared cases and what was reported in ours, that in patients who have an indication to perform bariatric surgery and present SIT, surgery is not contraindicated, obtaining favorable results.

## INTRODUCTION

Situs inversus is present in 0.01% of the population, which is transmitted by autosomal recessive genes. Situs describes the position of the cardiac atria and viscera [1, 2]. Situs solitus is the normal position, and situs inversus is the mirror image of situs solitus [3, 4]. Situs inversus can be classified further as situs inversus with levocardia and as situs inversus with dextrocardia [1]. Situs inversus with dextrocardia is also termed situs inversus totalis (SIT) because the cardiac position, as well as the atrial chambers and abdominal viscera, is a mirror image of the normal anatomy [5, 6]. SIT has an incidence of 1 in 8000 births [7, 8]. Situs inversus with levocardia is less common, with an incidence of 1 in 22 000 births [1].

## CASE REPORT

**Figure 1:**
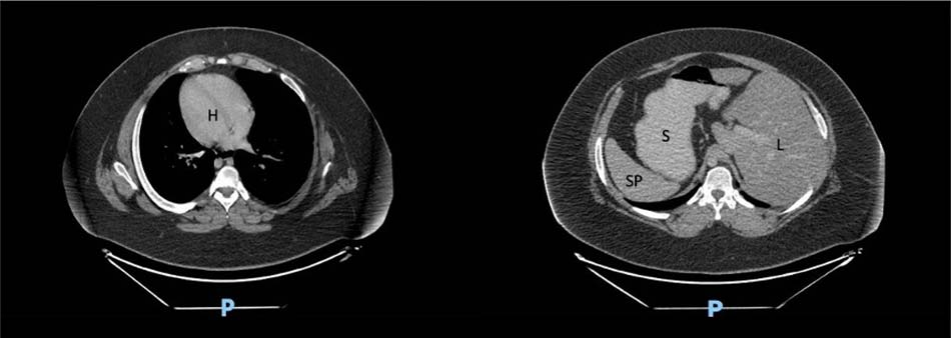
Computed tomography scan of the abdomen in our patient with SIT showing dextrocardia (H), spleen (SP) and stomach (S) located on the right side and liver (L) on the left side.

**Figure 2:**
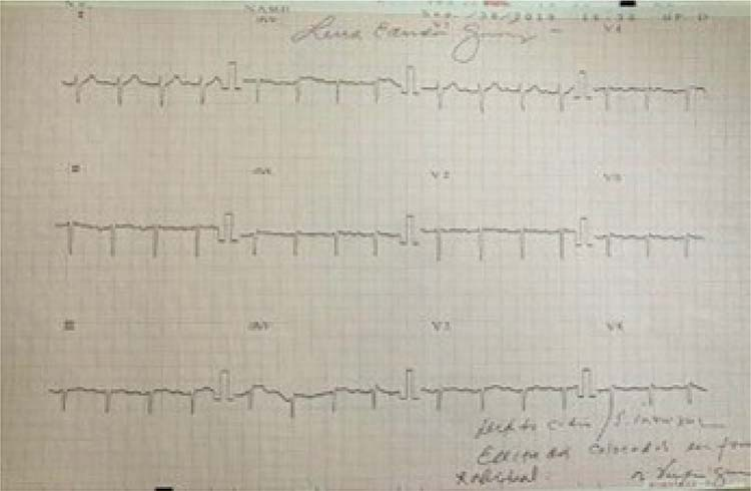
Electrocardiogram in dextrocardia; axis deviation to the right; positive QRS complexes (with positive P and T waves) in aVR; lead I: inversion of all complexes (inverted P wave, negative QRS, inverted T wave).

**Figure 3:**
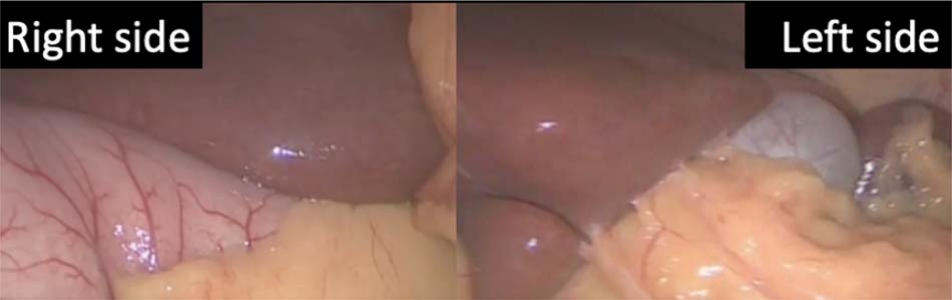
The lesser curvature of the stomach to the right of the falciform ligament, and the gallbladder located in the left upper quadrant.

We present the case of a 19-year-old male patient with a known preoperative diagnosis of SIT, which was discovered in childhood after diagnosis of a pleural effusion treated by placing a chest tube for drainage (Figs 1 and 2). The patient weighed 156 kg and was 177 cm tall, with a preoperative body mass index (BMI) of 49.8 kg/m2, without chronic-degenerative comorbidities. The patient failed to achieve weight loss despite multiple attempts at diet and exercise therapy. After performing a detailed preoperative evaluation, it was proposed to perform an Roux-en-Y laparoscopic gastric bypass (RYGB) for definitive weight loss. The patient is admitted to the operating room to perform the procedure with minimal adjustments in terms of surgical technique. The procedure is performed with the mirror technique from the conventional manner due to the patient’s baseline conditions (Fig. 3). Given the complexity of the case, the surgery was performed for a longer period of time with a total surgical time of 150 min. The patient was subsequently transferred to the recovery room without complications in the immediate or mediate post-operative period.

**Table 1:** Comparison of results in the reports of cases in RYGB in patients with SIT

Author	Year	Gender	Age	Duration	Complications	Hospital stay
Ahmed	2066	F	47	160 min	No	2 days
Wall (Robot)	2013	M	58	-	No	3 days
Stier	2014	M	39	76 min	No	5 days
Nelson	2016	F	43	-	No	2 days
Atwez	2018	F	43	180 min	No	2 days
Poghosyan	2020	-	58	130 min	No	3 days
Zorrila	2020	M	19	150 min	No	3 days

## SURGICAL TECHNIQUE

The surgical procedure begins by placing five trocars. Once the lens is inserted into the abdominal cavity, the stomach is identified to the right of the falciform ligament, and the gallbladder located in the upper left quadrant. As a first step, a 25-cc gastric pouch was made with a linear endostapler with a blue cartridge. Subsequently, a 140-cm bilio-pancreatic loop and a 60-cm Roux loop were made using a linear endostapler with a white cartridge and reinforcing with a vicryl suture to perform the jejuno-jejuno anastomosis. Finally, the gastro-jejunal anastomosis was manually performed in two planes with vicryl suture. The Petersen and mesenteric spaces were closed with ethibond suture. Finally, a closed Blake-type drain was placed and the abdominal wall was closed in layers.

### Surgery results

The patient had a favorable post-operative course and was discharged on the second post-operative day. He currently has done 2 years of post-operative follow-up, and his current BMI is 27 kg/m2, with a total weight loss of 71 kg.

## DISCUSSION

A search was made for case reports of bariatric surgery in patients with situs inversus in PubMed, finding six previously published cases of RYGB, and one of them assisted with a robot and published by Wall *et al*. The data compared for each case included: sex, age, BMI, technique, duration of surgery, complications, post-operative evolution and hospital stay (Table 1).

In the reported cases, equity is observed in terms of distribution by sex, with three cases reported in a female patient, two cases in a male and one case that was not specified. There is an age range of 39–58 years. Regarding the surgical time, they are between 76 and 180 minutes; in most cases, surgeons reported longer times than usual. No complication was reported in any of the cases. Hospitalization days have been reported from two to five days.

The age of our patient has been the lowest reported compared to the cases mentioned, and like the rest, the patient did not present any complications. Surgical time was similar to the reported average. Our patient was discharged home on his second post-operative day. The patient presented for follow-up, and 6 months later, was still with no complications.

## CONCLUSION

It can be concluded, based on the previously compared cases and what was reported in ours, that in patients who have an indication to perform bariatric surgery and present SIT, surgery is not contraindicated, obtaining favorable results. The prolongation of surgical times is not significant enough to put the patient at risk, and the length of hospital stay, as well as their recovery, has proven to be similar to those of other patients.
